# Cerebral Cortical Surface Structure and Neural Activation Pattern Among Adolescent Football Players

**DOI:** 10.1001/jamanetworkopen.2023.54235

**Published:** 2024-02-01

**Authors:** Taylor R. Zuidema, Jiancheng Hou, Kyle A. Kercher, Grace O. Recht, Sage H. Sweeney, Nishant Chenchaiah, Hu Cheng, Jesse A. Steinfeldt, Keisuke Kawata

**Affiliations:** 1 Department of Kinesiology, Indiana University School of Public Health, Bloomington; 2 Program in Neuroscience, The College of Arts and Sciences, Indiana University, Bloomington; 3 Research Center for Cross-Straits Cultural Development, Fujian Normal University, Fuzhou, Fujian, China; 4 Department of Psychological and Brain Sciences, College of Arts and Sciences, Indiana University, Bloomington; 5 Department of Counseling and Educational Psychology, School of Education, Indiana University, Bloomington; 6 Department of Pediatrics, Indiana University School of Medicine, Indianapolis

## Abstract

**Importance:**

Recurring exposure to head impacts in American football has garnered public and scientific attention, yet neurobiological associations in adolescent football players remain unclear.

**Objective:**

To examine cortical structure and neurophysiological characteristics in adolescent football players.

**Design, Setting, and Participants:**

This cohort study included adolescent football players and control athletes (swimming, cross country, and tennis) from 5 high school athletic programs, who were matched with age, sex (male), and school. Neuroimaging assessments were conducted May to July of the 2021 and 2022 seasons. Data were analyzed from February to November 2023.

**Exposure:**

Playing tackle football or noncontact sports.

**Main Outcomes and Measures:**

Structural magnetic resonance imaging (MRI) data were analyzed for cortical thickness, sulcal depth, and gyrification, and cortical surface-based resting state (RS)–functional MRI analyses examined the amplitude of low-frequency fluctuation (ALFF), regional homogeneity (ReHo), and RS-functional connectivity (RS-FC).

**Results:**

Two-hundred seventy-five male participants (205 football players; mean [SD] age, 15.8 [1.2] years; 5 Asian [2.4%], 8 Black or African American [3.9%], and 189 White [92.2%]; 70 control participants; mean [SD] age 15.8 [1.2] years, 4 Asian [5.7], 1 Black or African American [1.4%], and 64 White [91.5%]) were included in this study. Relative to the control group, the football group showed significant cortical thinning, especially in fronto-occipital regions (eg, right precentral gyrus: *t* = −2.24; *P* = .01; left superior frontal gyrus: −2.42; *P* = .002). Elevated cortical thickness in football players was observed in the anterior and posterior cingulate cortex (eg, left posterior cingulate cortex: *t* = 2.28; *P* = .01; right caudal anterior cingulate cortex 3.01; *P* = .001). The football group had greater and deeper sulcal depth than the control groups in the cingulate cortex, precuneus, and precentral gyrus (eg, right inferior parietal lobule: *t* = 2.20; *P* = .004; right caudal anterior cingulate cortex: 4.30; *P* < .001). Significantly lower ALFF was detected in the frontal lobe and cingulate cortex of the football group (*t* = −3.66 to −4.92; *P* < .01), whereas elevated ALFF was observed in the occipital regions (calcarine and lingual gyrus, *t* = 3.20; *P* < .01). Similar to ALFF, football players exhibited lower ReHo in the precentral gyrus and medial aspects of the brain, such as precuneus, insula, and cingulum, whereas elevated ReHo was clustered in the occipitotemporal regions (*t* = 3.17; *P* < .001; to 4.32; *P* < .01). There was no group difference in RS-FC measures.

**Conclusions and Relevance:**

In this study of adolescent athletes, there was evidence of discernible structural and physiological differences in the brains of adolescent football players compared with their noncontact controls. Many of the affected brain regions were associated with mental health well-being.

## Key Points

**Question:**

Is playing tackle football associated with brain surface structure and functional alterations in adolescent football players?

**Findings:**

In a cohort study of 205 adolescent football players and 70 noncontact control athletes, adolescent football players showed signs of altered brain structures including cortical thinning and changes in brain folding. Also, football players showed lower brain signaling and coherence in frontal and medial parts of the brain, but increased signaling and coherence were observed in the occipital lobe.

**Meaning:**

These findings suggest playing football may be associated with a different trajectory of cortical maturations and aging processes, and many affected brain regions are associated with mental well-being.

## Teaser

This cohort study evaluates cortical structure and neurophysiological characteristics in adolescent football players compared with other athletes.

## Introduction

A single football possesses the extraordinary ability to foster unity among individuals spanning diverse ages, races and ethnicities, and socioeconomic backgrounds.^1^ However, this unifying force comes at a cost, with repetitive subconcussive head impacts often stemming from tackling, posing a risk of triggering neurodegenerative disorders.^2^ This issue is particularly pertinent to young athletes, as a recent study^3^ has reported cases of chronic traumatic encephalopathy among young adults with a history of head impact exposure.

Numerous neuroimaging studies have delved into the neurological correlates of sports-related brain injuries. For example, former athletes engaged in collision sports (eg, football, soccer, boxing) exhibit reduced cortical thickness across widespread brain regions compared with their nonathlete counterparts.^4,5,6^ A large-scale pediatric mild traumatic brain injury (mTBI) study revealed significant cortical thinning in frontal and occipital regions among male patients with persistent symptoms beyond 3 months compared with those without persistent symptoms.^7^ Likewise, current high school and college football players showed greater brain volume reduction and cortical thinning in frontotemporal regions after a single^8^ and also several seasons,^9^ compared with control athletes (eg, volleyball). Furthermore, resting-state (RS) functional magnetic resonance imaging (fMRI) functional connectivity has revealed neurophysiological changes due to repetitive head impacts. For instance, decreased connectivity was pronounced in frontal and cingulate regions, whereas increased connectivity was identified in posterior brain regions (eg, precuneus, supramarginal gyrus) after a rugby game^10^ and a season in collision sports.^11,12,13^ However, limitations in prior studies, such as small sample sizes from a single high school program and narrow panels of imaging, hinder the broad implications of these findings.

Addressing these limitations, multiparameter morphometric techniques, encompassing volumetric analysis (cortical thickness) and geometric analysis (gyrification and sulcal depth), provide valuable insights into the neurobiological underpinnings of maturation, aging, injury, and degenerative conditions.^14,15^ The cortical thickness informs changes in gray matter volumes, whereas the gyrification and sulcal depth examine the complexity and depth of cortical folding, respectively.^16^ In addition, a cortical surface-based fMRI analysis^17^ offers a novel approach to examine not only the RS functional connectivity (FC) but also the amplitude of low-frequency fluctuation (ALFF) and regional homogeneity (ReHo) on the cortical surface.^18^ ALFF estimates the total neural signaling power, while ReHo gauges the coherence of neighboring neural activity.

In this cohort study, we applied these advanced neuroimaging techniques to uncover cortical structure and neurophysiological differences between high school football players and controls participating in noncontact sports. Our hypothesis was that compared with controls, the football group would exhibit (1) lesser cortical thickness and gyrification and greater sulcal depth, (2) lower localized neural activity (ALFF) and coherence of neural signals (ReHo) and (3) altered RS-FC involving the dorsolateral prefrontal cortex (DLPFC). The selection of the DLPFC as a seed is grounded in its role as a neural network hub, supported by previous studies demonstrating altered activation patterns in the DLPFC after a football season^19^ and the presence of neurofibrillary tangle in this region among young adults with chronic traumatic encephalopathy.^3^

## Methods

### Participants

This cohort study included male high school football players and male high school noncontact control athletes from 5 high schools in the Midwest. The data collection was conducted during preseason before the 2021 and 2022 seasons. Inclusion criteria were being current members of the high school football team or noncontact sports team (swimming, cross country, and tennis) and being between the ages of 13 and 18 years. Control participants were matched to football participants with sex (male), age, and school they attend. Exclusion criteria were a history of moderate-to-severe TBI, organized contact sports experience for the control athletes, and any MRI contraindications. A history of mTBI or concussion was permitted if asymptomatic for the past 6 months. Race and ethnicity data were based on self-report and were assessed in this study because providing racial data will help interpret the neurological outcomes in relation to head impact burden from football participation. All participants and their legal guardians provided informed consent, and the Indiana University institutional review board approved the study protocol. This study followed the Strengthening the Reporting of Observational Studies in Epidemiology (STROBE) reporting guideline.

### MRI Data Acquisition

The MRI data were acquired on a 3T Prisma MRI scanner (Siemens) equipped with a 64-channel head/neck coil. Cortical morphometry measures were based on high-resolution anatomical images (T1-weighted) acquired using 3D MPRAGE pulse sequence with the following parameters: repetition time (TR) and echo time (TE), 2400/2.3 ms; inversion time, 1060 ms; flip angle, 8; matrix, 320 × 320; bandwidth, 210 Hz/pixel; integrated parallel acquisition technique, 2, which resulted in 0.8-mm isotropic resolution. Resting-state blood oxygen level–dependent (BOLD) signal was collected using a simultaneous multislice, single-shot echo-planar imaging sequence: TR/TE, 800/30 ms; flip angle, 52°; matrix, 90 × 90; field-of-view, 216 mm; resolution, 2.4 mm isotropic; and multiband acceleration factor, 6, with 1000 total volumes acquired over 12 minutes while the participant passively viewed a crosshair.

### Cortical Morphometry Preprocessing and Analyses

The detailed preprocessing workflow is described in the eMethods in Supplement 1. The Computational Anatomy Toolbox 12v (CAT12) version 6 (Structural Brain Mapping Group) was used for the T1-weighted MRI data preprocessing. The preprocessing consisted of bias-field correction, skull-stripping, and alignment to the Montreal Neurological Institute structural template to classify gray matter (GM), white matter (WM), and cerebrospinal fluid (CSF). Spatial normalization was conducted with the Diffeomorphic Anatomical Registration Through Exponentiated Lie Algebra registration (1.5 mm).^20^ A spherical harmonic method was used to reparametrize the cortical surface mesh.^20,21^ A voxel-based distance method was used to estimate the WM segment by calculating the distance from the inner GM boundary. Values at the outer GM boundary in the WM distance map were projected back to the inner GM boundary to generate the GM thickness. The cortical thickness data were spatially smoothed with a Gaussian kernel with a 15 mm full-width at half-maximum (FWHM). The gyrification estimates cortical fold complexity based on spherical harmonics and was calculated as absolute mean curvature.^22^ The sulcal depth is calculated as the Euclidean distance between the central surface and its convex hull based on the spherical harmonics, then transformed with the sqrt function.^22^

### Preprocessing for Surface-Based RS-fMRI Analysis

The workflow of the preprocessing for the surface-based RS-fMRI analysis is extensive, as described in the eMethods in Supplement 1. The brief summary of steps is as follows: (1) a reference volume and its skull-stripped version were generated using fMRIPrep; (2) the BOLD reference was coregistered to the T1 image; (3) participants with head motions beyond a frame-wise displacement of more than 3.0 mm and 3.0 degrees were excluded from fMRI analyses; and (4) the BOLD time-series were resampled onto their original, native space.

### ALFF and ReHo Analyses

ALFF and ReHo analyses were conducted using DPABISurf version 1.2 (R-fMRI Network).^17,23^ For ALFF, the resampled functional images were spatially smoothed with a FWHM of 6 mm. The square root of the power spectrum was calculated to obtain a raw ALFF map. ALFF values for each voxel were divided by the global mean ALFF value for standardization. Surface-based ReHo maps were produced by calculating the concordance of the Kendall coefficient of the time series of a given vertex in the surface space with nearest neighbors. This computational approach was repeated for all vertices in surfaces. The individual ReHo maps were smoothed by a 3D Gaussian kernel of 6 mm FWHM for further statistical analysis.

### Analyses for Seed-Based RS-FC Analysis

As with ALFF and ReHo, the seed-based FC analyses were conducted on the cortical surface. First, temporal filtering was applied to the fMRI data, specifically in the frequency range of 0.01 to 0.1 Hz. The mean CSF signal was removed by linear regression. Then, the 3-dimensional volumes comprising 210 images for each participant were transformed to the surface. Normalization procedures, including a smoothing operation within the surface space to a 9-mm radius, were performed. For the surface mapping of the fMRI data, a weighted-mean method that uses a Gaussian kernel for mapping along the normal was applied. Lastly, regions of interest extraction was performed within the surface space. The bilateral DLPFC was selected as our region-of-interest (ROI) because of its involvement in brain injury.^24^ A Pearson correlation coefficient was generated between the DLPFC and the whole brain. The correlation coefficients were transformed into Fisher *z* scores for statistical analysis.

### Statistical Analysis

Demographic differences between the football and control groups were assessed by 2-sample *t* tests for continuous variables and χ^2^ tests for categorical variables. Mann-Whitney *U* test was used for General Anxiety Disorder-7 (GAD-7) and Patient Health Questionnaire-9 (PHQ-9) due to significant results in Shapiro-Wilk normality tests. Group comparison on morphological metrics was performed using CAT12 and analyzed via a nonparametric permutation technique (5000 permutations). Age, body mass index (BMI), number of previous concussions, depression score via PHQ-9, anxiety score via GAD-7, and intracranial volume were included as covariates. The threshold-free cluster enhancement (TFCE) was used in the permutation test, which gives cluster-based thresholding for familywise error correction, and the level of significance was set to *P* < .02 to reflect 3 morphological outcomes.

For the RS-fMRI analyses, the 2-tailed, independent-samples *t* tests were performed in the DPABISurf toolkit to compare group differences in mean and peak ALFF and ReHo. For ALFF and ReHo, the statistical significance was set to *P* < .01. We used TFCE to obtain a significant group difference. Similarly, the 2-tailed, independent-samples *t* tests were used to compare RS-FC strengths between groups. For all RS-fMRI analyses, age, BMI, previous concussions, and mean head motion were included as covariates. Multiple comparisons were corrected by TFCE, and the level of statistical significance was set at 2-tailed *P* < .03 to reflect bilateral DLPFC as seeds. The number of permutations was set at 5000. Data were analyzed from February to November 2023.

## Results

### Demographic Characteristics

A total of 275 participants (205 football players and 70 noncontact athletes in the control group) were included in this study. The sample consisted of all males: 2 (1%) were American Indian or Alaska Native; 9 (3%) were Asian; 9 (3%) were Black or African American; 2 (1%) were multiracial; and 253 (92%) were White. A total of 23 participants (8%) were Latino or Hispanic, and 252 (92%) were not Latino or Hispanic. Demographics are summarized in Table 1. All samples contributed to the cortical structure analysis. For RS-fMRI analysis, data from 56 football participants and 11 controls were excluded due to head motion exceeding a frame-wise displacement of 3.0 mm. As a result, a total of 208 participants (149 football players and 59 in the control group) contributed to the RS-fMRI analyses.

**Table 1.** Demographic Characteristics Between Groups

Variable	Participants, No. (%)		*P* value
	Football (n = 205)	Control (n = 70)	
Male sex	205 (100)	70 (100)	NA
Age, mean (SD), y	15.8 (1.21)	15.8 (1.2)	.217
Body mass index, mean (SD)^a^	26.9 (5.9)	22.0 (6.2)	<.01
Race			
American Indian or Alaska Native	1 (0.5)	1 (1.4)	.21
Asian	5 (2.4)	4 (5.7)	
Black or African American	8 (3.9)	1 (1.4)	
White	189 (92.2)	64 (91.5)	
Multirace	2 (1.0)	0 (0.0)	
Ethnicity			
Not Latino or Hispanic	189 (92.2)	63 (90.0)	.32
Latino or Hispanic	16 (7.8)	7 (10.0)	
No. of previous concussions			
0	150 (73.2)	57 (88.6)	.05
1	45 (21.9)	7 (10.0)	
2	10 (4.9)	1 (1.5)	
Years of tackle football experiences, mean (SD)	5.6 (8.8)	0	NA
Age of first exposure to tackle football, mean (SD), y	10.9 (3.0)	0	NA
PHQ-9 score, mean (SD)	2.67 (3.8)	2.7 (2.7)	.12
GAD-7 score, mean (SD)	2.0 (3.0)	2.5 (3.3)	.89

Abbreviations: GAD-7, General Anxiety Disorder-7 (assess anxiety symptoms); NA, not applicable; PHQ-9, Patient Health Questionnaire-9 (assesses depressive symptoms).

^a^ Body mass index is calculated as weight in kilograms divided by height in meters squared.

### Group Differences in Cortical Structure

**Figure 1.**
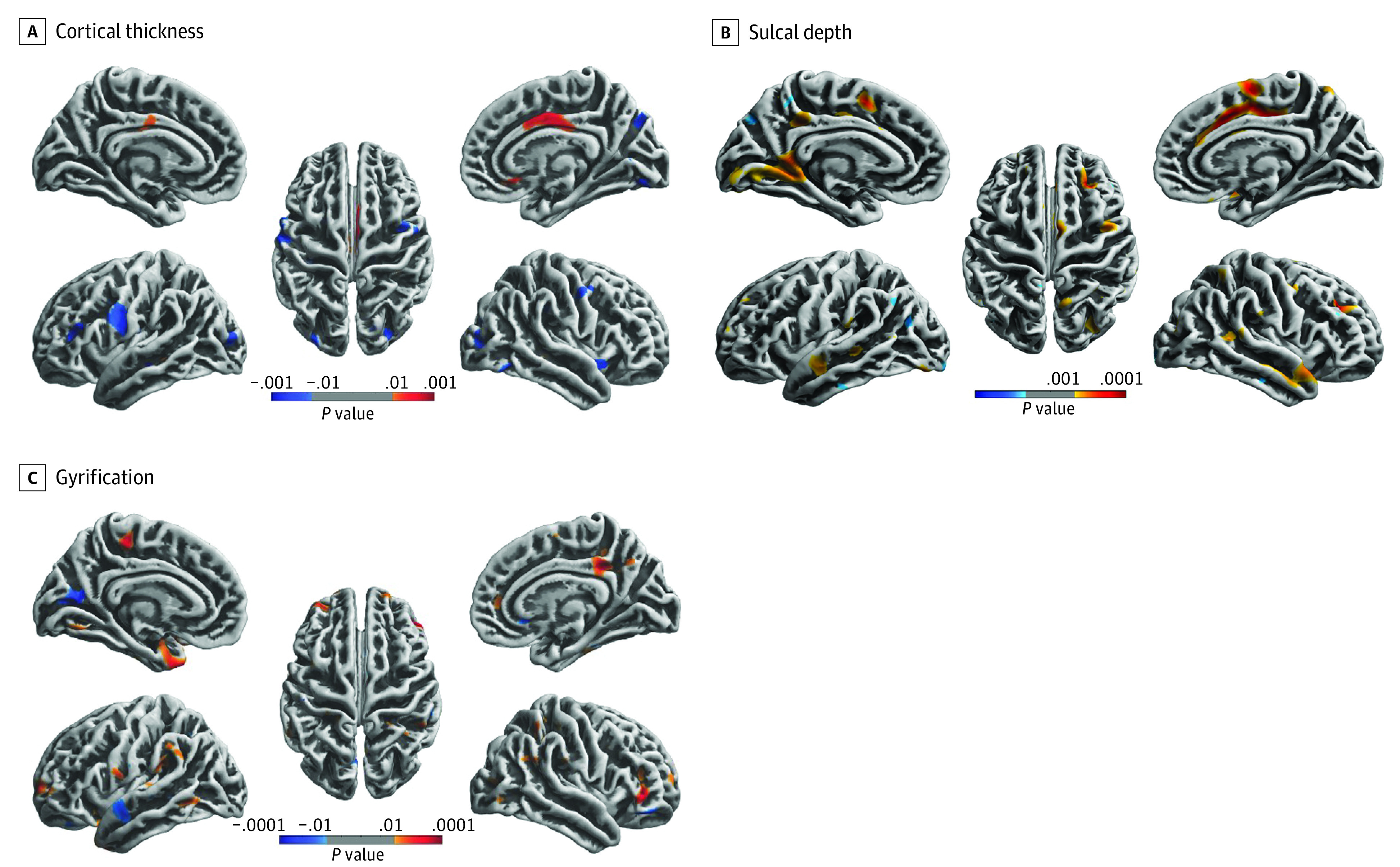
Cortical Morphological Differences Between the Adolescent Football Players and Control Athletes Group difference was assessed for cortical thickness (A), sulcal depth (B), and gyrification (C). The multiple comparison correction was used with nonparametric permutations (5000 permutations) and the threshold-free cluster enhancement correction after 5000 permutations. Red indicates that the football group is higher compared with the control group. Blue indicates that the football group is lower compared with the control group.

Relative to the control group, the football group showed significant cortical thinning in various brain regions in both hemispheres, such as precentral gyrus, lateral occipital gyrus, lingual gyrus, and middle or superior frontal gyrus (eg, right precentral gyrus: *t* = −2.24; *P* = .01; left superior frontal gyrus: −2.42; *P* = .002) (Figure 1A). Conversely, there was a notable thickening in the anterior and posterior cingulate cortex in the football group relative to controls (eg, left posterior cingulate cortex: *t* = 2.28; *P* = .01; right caudal anterior cingulate cortex 3.01; *P* = .001).

A significantly greater (deeper) sulcal depth was observed in widespread brain regions of the football group compared with controls, such as the cingulate cortex, precentral gyrus, and frontotemporal regions (eg, right inferior parietal lobule: *t* = 2.20; *P* = .004; right caudal anterior cingulate cortex: 4.30; *P* < .001) (Figure 1B). Despite small clusters, there were 4 regions showing lesser (shallower) sulcal depth in the football group, including left temporal pole, superior parietal lobule, pericalcarine gyrus, and lateral occipital gyrus (eg, left pericalcarine gyrus: *t* = −2.13; *P* = .01; left temporal pole: 3.40; *P* = .01).

Compared with the control group, the football group showed greater gyrification in many regions in both hemispheres, such as the cingulate cortex, frontoparietal regions, precuneus, and lingual gyrus (eg, left lingual gyrus: *t* = 2.44; *P* = .01; right posterior cingulate cortex: *t* = 3.44; *P* < .001), whereas lesser gyrification was observed in the pericalcarine, superior temporal gyrus, parsobitalis gyrus, and caudal anterior cingulate cortex (eg, left superior temporal gyrus: *t* = −2.38; *P* = .004; left pericalcarine gyrus: −3.15; *P* < .001) (Figure 1C). Detailed information for each brain region is listed in Table 2.

**Table 2.** Differences in Cortical Structure Between the Football and Control Groups

Measure and brain regions	BA	Coordinates			Cluster size	Peak *t* value^a^	*P* value
		*x*	*y*	*z*			
Cortical thickness							
Football > control							
Left posterior cingulate cortex	23	−12	−11	46	59	2.28	.01
Right posterior cingulate cortex	23	3	−12	33	229	2.95	.001
Right caudal anterior cingulate cortex	24	3	−2	40	51	3.01	.001
Football < control							
Left rostral middle frontal gyrus	11	−4	61	−10	84	−2.38	.002
Left superior frontal gyrus	9	−4	−11	30	66	−2.42	.002
Left lateral occipital gyrus	17	−3	−87	−7	54	−2.33	.004
Right lateral occipital gyrus	17	14	−83	8	89	−2.40	.004
Right lingual gyrus	19	31	−83	−15	72	−2.33	.001
Right fusiform	18	23	−58	−8	37	−2.41	.004
Right insula	47	43	16	−6	55	−2.26	.01
Right precuneus	7	5	−58	39	31	−2.31	.01
Right precentral gyrus	6	17	−19	68	43	−2.24	.01
Right inferior temporal gyrus	21	47	−35	−4	31	−2.39	.004
Sulcal depth							
Football > controls							
Left lingual gyrus	19	−35	−85	−18	290	3.14	.001
Left isthmus cingulate cortex	23	−10	−8	42	224	2.62	.001
Left precuneus	30	−7	−49	7	80	3.13	.001
Left superior frontal gyrus	11	−18	45	38	78	2.29	<.001
Left superior temporal gyrus	21	−52	−34	−2	75	2.82	.003
Left posterior cingulate cortex	24	−13	−43	36	69	3.18	.001
Left lateral occipital gyrus	17	−5	−88	−2	46	2.40	.001
Left bankssts gyrus	42	−60	−29	18	42	2.40	.003
Right superior frontal gyrus	6	7	−4	62	459	4.06	<.001
Right superior temporal gyrus	21	49	7	−17	268	3.25	.001
Right middle temporal gyrus	20	58	−12	−19	171	2.94	.001
Right rostral middle frontal gyrus	44	43	4	30	165	2.42	<.001
Right superior parietal lobule	40	35	−51	57	149	2.56	.002
Right posterior cingulate cortex	7	14	−20	40	137	4.23	<.001
Right paracentral gyrus	7	10	−63	63	129	2.50	<.001
Right caudal anterior cingulate cortex	32	11	13	41	81	4.30	<.001
Right precentral gyrus	6	43	0	46	81	3.00	.002
Right bankssts gyrus	21	51	−46	7	47	2.65	.004
Right inferior parietal lobule	48	60	−25	27	40	2.20	.004
Football < controls							
Left lateral occipital gyrus	19	−35	−85	−18	30	−2.36	.01
Left superior parietal lobule	5	−11	−58	56	72	−2.71	.001
Left pericalcarine gyrus	19	25	−81	17	35	−2.13	.01
Left temporal pole	36	−27	12	−40	21	3.40	.01
Gyrification							
Football > controls							
Left temporal pole	36	−27	12	−40	86	3.40	<.001
Left supramarginal gyrus	40	−51	−35	25	123	2.93	.002
Left paracentral gyrus	4	−5	−28	54	88	3.42	<.001
Left rostral middle frontal gyrus	10	−21	62	6	84	3.05	.002
Left lateral orbitofrontal gyrus	11	−25	42	−11	52	3.28	.001
Left rostral middle frontal gyrus	11	−48	−23	8	35	2.64	.001
Left inferior temporal gyrus	37	−55	−56	−5	60	2.91	.002
Left lingual gyrus	19	−31	−75	−18	50	2.44	.01
Right isthmus cingulate cortex	23	17	−42	47	59	2.74	<.001
Right posterior cingulate cortex	23	9	−39	36	31	3.44	<.001
Right parstriangularis gyrus	45	46	37	3	98	3.17	<.001
Right precuneus	7	5	−62	40	45	2.61	<.001
Right rostral middle frontal gyrus	45	47	37	3	73	3.43	<.001
Right superior frontal gyrus	9	15	34	51	63	2.56	.01
Right inferior parietal lobule	40	35	−49	51	78	2.88	.003
Right superior parietal lobule	6	25	−42	59	46	3.11	.002
Right supramarginal gyrus	40	51	−43	43	41	2.81	.002
Football < controls							
Left pericalcarine gyrus	17	−17	−56	6	110	−3.15	<.001
Left superior temporal gyrus	48	−47	−26	8	69	−2.38	.004
Right parsorbitalis gyrus	10	7	47	−5	76	−2.59	<.001
Right caudal anterior cingulate cortex	32	15	43	9	30	−2.62	<.001

Abbreviation: BA, Brodmann area.

^a^ The multiple comparison correction was used by the threshold-free cluster enhancement correction. Positive *t* values indicate the football group is higher compared with the control group, and negative *t* values indicate the football group is lower compared with the control group.

### Group Differences in ALFF and ReHo

**Figure 2.**
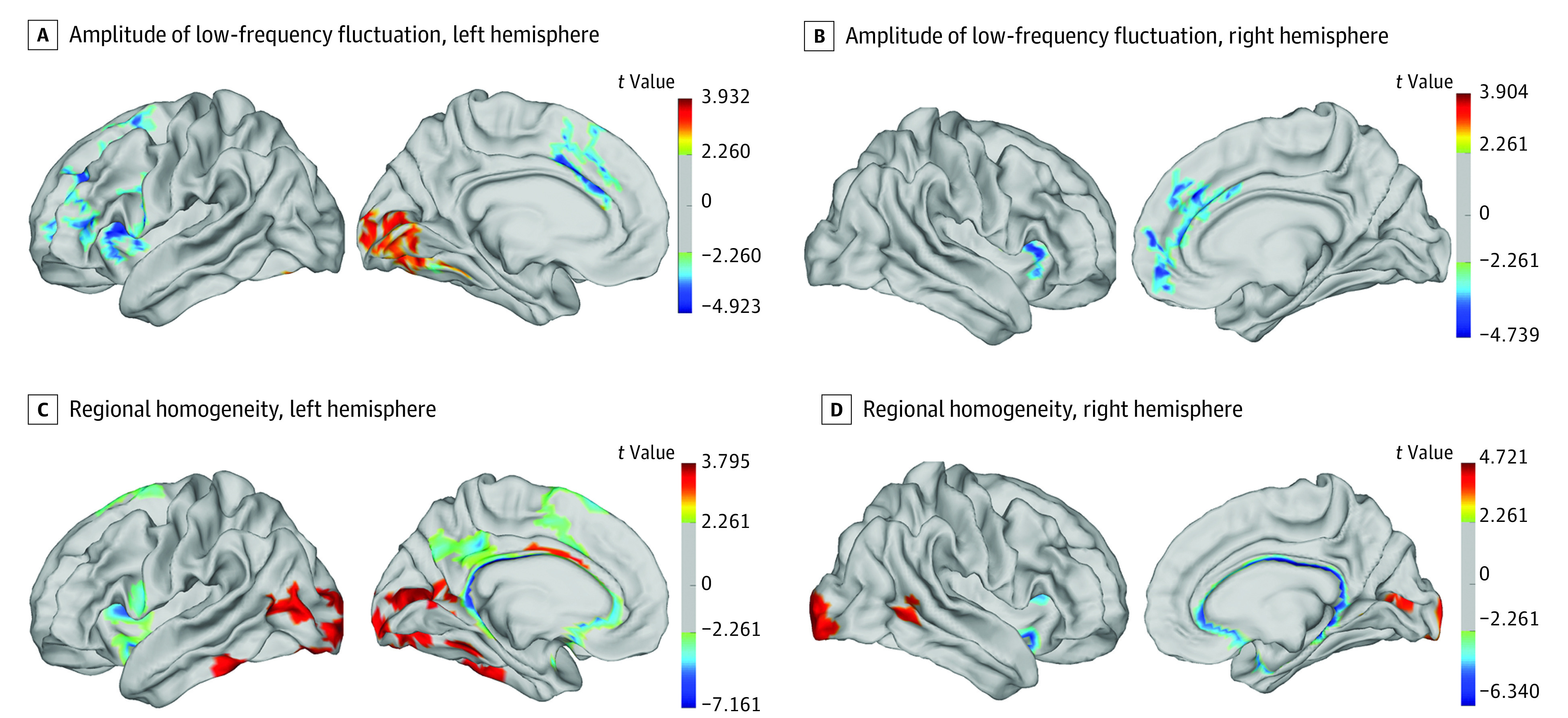
Regional Differences in Neurophysiology Between the Football and Control Groups The surface-based resting state–functional magnetic resonance imaging technique was used to test the group differences in amplitude of low-frequency fluctuation (A and B) and regional homogeneity (C and D). The multiple comparison correction was used with nonparametric permutations (5000 permutations) and the threshold-free cluster enhancement correction after 5000 permutations.

Significantly lower ALFF was detected in large areas of the frontal regions and cingulate cortex in the football group relative to the control group, such as left middle, superior, and triangular frontal gyri; left precentral gyrus; middle and anterior cingulate cortex; and bilateral insula (*t* = −3.66 to −4.92; *P* < .01) (Figure 2A, 2B). Conversely, significantly higher ALFF was noted in the left medial occipital regions of the football group, including the lingual gyrus (*t* = 3.20; *P* < .01) and calcarine sulcus (*t* = 3.28; *P* < .01).

Similar to ALFF, there was a significantly higher ReHo in the occipitotemporal regions of the football group relative to the control group, including lingual, inferior and middle occipital gyri, calcarine sulcus, and middle and inferior temporal gyrus (*t* = 3.17 to 4.32; *P* < .01). Significantly lower ReHo was observed in the bilateral precentral gyri, as well as medial brain regions, including bilateral middle and posterior cingulate cortex, insula and putamen (*t* = −3.65 to *t* = −7.16) (Figure 2C, 2D). Detailed information for each brain region is listed in Table 3.

**Table 3.** Differences in Amplitude of Low-Frequency Fluctuation and Regional Homogeneity Between the Football and Control Groups

Measure and brain region	BA	Coordinates			Cluster size	Peak *t* value^a^
		*x*	*y*	*z*		
ALFF						
Football > control						
Left calcarine	17	−5	−99	8	85	3.201
Left lingual gyrus	18	−20	−75	5	115	3.278
Football < control						
Left precentral gyrus	6	−50	3	17	208	−4.923
Left middle frontal gyrus	46	−25	42	17	296	−4.429
Left middle frontal gyrus	8	−24	14	48	208	−3.863
Left superior frontal gyrus	11	−23	51	1	69	−4.024
Left inferior triangular frontal gyrus	45	−47	33	8	117	−3.655
Left insula	48	−30	17	3	170	−4.798
Left middle cingulate cortex	32	−11	7	39	289	−4.359
Right anterior cingulate cortex	32	11	33	30	418	−4.739
Right insula	48	34	6	7	140	−4.332
ReHo						
Football > control						
Left lingual gyrus	18	−15	−77	11	744	3.564
Left inferior occipital gyrus	17	−23	−98	−4	526	3.486
Left middle occipital gyrus	37	−44	−62	5	351	3.205
Left middle occipital gyrus	18	−28	−94	13	173	3.174
Left inferior temporal gyrus	20	−54	−43	−23	256	3.512
Left fusiform	37	−28	−49	−15	109	3.795
Right calcarine	17	17	−102	−3	464	4.317
Right calcarine	17	16	−81	8	176	3.302
Right middle temporal gyrus	21	59	−52	−2	133	3.476
Football < control						
Left middle cingulate cortex	32	−10	14	33	505	−3.716
Left posterior cingulate cortex	23	−5	−32	26	947	−7.161
Left precuneus	48	−31	12	3	653	−5.181
Left precentral gyrus	6	−50	3	19	245	−4.185
Right posterior cingulate cortex	29	4	−31	26	300	−6.340
Right insula	48	36	5	−17	75	−4.604
Right putamen	48	32	10	8	107	−3.671
Right precentral gyrus	6	34	−8	49	61	−3.650

Abbreviation: BA, Brodmann area.

^a^ The multiple comparison correction was used by the threshold-free cluster enhancement correction. Positive *t* values indicate the football group is higher compared with the control group, and negative *t* values indicate the football group is lower compared with the control group.

### Group Difference in RS-FC

RS-FC was also assessed on the cortical surface, and the DLPFC was our seed region due to its role as a network hub and one of the regions vulnerable to brain trauma. Our analysis revealed that there were no significant differences in functional connectivity (*t* < 2.0, *P* > .05).

## Discussion

The current study revealed distinctive neuroanatomical and physiological attributes associated with high school football players, delineating 4 key findings. First, these players displayed cortical thinning in numerous localized areas of the frontal and occipital regions, alongside cortical thickening in the cingulate cortex. Second, an increase in sulcal depth and greater gyrification manifested across various brain regions in football players. Third, an intriguing pattern of local neural activity (ALFF) emerged, revealing decreased ALFF in the frontal region and increased ALFF in the occipital region among football players. Lastly, coherence of neural signals (ReHo) was similar with the ALFF findings, suggesting decreased ReHo in the frontal and medial regions (eg, cingulate, insula) and increased ReHo in the occipitotemporal regions. These data collectively suggest the presence of discernible structural and physiological differences in the brains of adolescent football players compared with their noncontact control counterparts.

The associations between repetitive head impacts and changes in axonal microstructural integrity have been documented in contact sports athletes,^25,26^ but changes in brain structure at a macro level are thought to require years of exposure to head impacts. Indeed, studies have reported cortical thinning in retired football and soccer players, specifically in the frontal, temporal, and parietal lobes.^4,5,6^ Yet, several studies argue that such a morphological change is absent in young adult soccer and rugby players aged 20 to 39 years.^27,28^ In contrast, our data showed significant cortical thinning in the fronto-occipital regions of adolescent football players’ brains, accompanied by cortical thickening in the cingulate cortex. TBI and repetitive exposure to head impacts can emerge as potential accelerators of age-related cortical thinning in these identified regions. Additionally, our observations align with a meta-analysis suggesting increased cortical thickness in the anterior/posterior cingulate cortex among medication-free patients with major depressive disorder.^29^ While group differences in PHQ-9 and GAD-7 were not significant in our samples, the cortical thickening observed in the cingulate cortex of football players introduces a new dimension to the discussion on the brains and mental health of adolescent football players. This is particularly relevant considering the frequent comorbidity of mental health disorders in trauma-induced neurodegenerative diseases.^30^

Our geometric analysis also revealed novel insights. The continual enlargement and maturation of cortical gyri and sulci are intrinsic to the developmental trajectory from youth through adolescence. While deepening and widening sulci are normal aspects of the aging process,^31^ these morphological changes are often accelerated in patients with neurodegenerative and psychiatric disorders.^32,33,34,35^ In our sample, greater sulcal depth was notable in football players, especially in the superior/middle frontal gyri and cingulate regions, which govern complex movement and the default mode network. Several computational studies suggest that the greatest tissue deformation occurs in the deepest sulci upon traumatic insult^36^ due to the cerebral spinal fluid rapidly clustering in the base of the sulci and causing shearing waves, referred to as water hammer effect.^31^ This type of mechanical stress can lead to accelerated tissue atrophy and deepening sulci.^36^ These findings shed light on the potential impact of mechanical stressors on brain structure and provide valuable insights into the dynamics of cortical changes in individuals engaged in football.

Although lower gyrification is often associated with cognitive impairment in older adults,^37^ a study by Wilde et al^38^ that included 17 adolescents with a history of TBI found significantly greater gyrification in the frontal and temporal regions compared with their controls. The researchers speculated the increased gyrification was part of a compensatory mechanism to bolster impaired brain regions.^38^ Our data are partly in agreement with Wilde et al^38^ in that many frontotemporal regions showed greater gyrification in the football group relative to the control group, whereas lesser gyrification was notable in parieto-occipital regions.

Our multiparameter fMRI approach uncovered physiological characteristics related to adolescent football players. We observed lower ALFF in widespread frontal regions in football players relative to controls, suggesting a lower density of neuronal signaling. Conversely, an elevated ALFF was observed in occipital regions, especially the calcarine sulcus and lingual gyrus, which are known for their involvement in visual memory and attention. These observations are in line with Xiong et al^39^ in that patients with concussions during the subacute phase of recovery exhibit a lower neural signaling density in the thalamus and frontal and temporal lobes, whereas increased ALFF is noted in the occipital regions. The observed ALFF patterns were mirrored in the ReHo results. Typically, the highest values of ReHo are found in the default-mode network (DMN) regions, including the prefrontal cortex, cingulum, precuneus, and angular gyrus, suggesting highly connected cortical hubs.^40^ However, significantly lower ReHo (football < control) was observed in several of the DMN regions, such as the cingulate cortex and precuneus, alongside medial brain regions such as the insula and putamen. Given that ReHo has demonstrated correlations with glucose (r = 0.78) and oxygen (r = 0.54) metabolic rates,^41^ the DMN in football players may exhibit lower coherence and regional metabolism. In contrast to the DMN findings, numerous cortical areas in the occipitotemporal regions displayed higher ReHo in the football group compared with the control group. This aligns with previous observations of increased ReHo in patients with acute concussions^42^ and those with persistent symptoms beyond 3 months after concussion.^43^ However, the clinical implications of these ReHo variations, beyond the coherence of neighboring neural connections and regional metabolism, warrant further investigation through well-controlled longitudinal studies.

Smaller-scale studies suggest that the network connectivity increases in the frontal and cingulate regions and decreases in posterior brain regions acutely after a rugby game^10^ and a season in collision sports.^11,12,13^ Hypoconnectivity stemming from the right DLPFC was notable in patients with concussions during memory and attention tasks.^44^ Unlike previous studies, we were unable to observe a group difference in functional connectivity with DLPFC as a seed. It is possible that since the DLPFC is a highly important brain area, impairments in this region due to concussive or subconcussive head impacts may be transient in nature. Thus, at preseason baseline, there were no discernible differences in functional connectivity in the DLPFC between football players and control athletes.

### Limitations

This study has limitations. While this is one of the largest neuroimaging studies of adolescent football players, more racial and ethnic diversity would be ideal. A lack of objective measures of history of head impact exposure and severities of head impacts is a limitation. The current study using a multimodal neuroimaging approach has provided a foundational basis that can be leveraged in a future longitudinal study, yet correlations between morphological and physiological changes should be investigated further. Additionally, it is imperative to integrate other neurological variables, such as psychometric assessments, blood biomarkers, and functional assessments, to holistically understand the relationships between head impact exposure and brain health in adolescent athletes.

## Conclusion

Our data derived from advanced neuroimaging techniques suggest that adolescent football players had reduced cortical thickness in fronto-occipital regions and increased thickness in the cingulate cortex, alongside deepened sulcal depth and altered gyrification in widespread regions. Football players also exhibited a lower density of neural activity and coherence of neural signaling in the frontal and medial regions of the brain, but increased activity and coherence were observed in the occipitotemporal regions. Many of the affected brain regions, especially reduced cortical thickness, increased sulcal depth, and reduced neural activity and coherence, were observed in brain areas that are important for mental health well-being. These data support the use of these imaging parameters in a future longitudinal study to gauge the subtle yet cumulative changes in brain structure and neurophysiological effects due to repetitive head impacts.

## Notes

Supplement 1. **eMethods.** Cortical Morphometry and Cortical Surface Functioning Preprocessing Workflow
eReferences

Supplement 2.
Data Sharing Statement
