# Supplementary material for: Cerebral Cortical Surface Structure and Neural Activation Pattern Among Adolescent Football Players
Source: JAMA Netw Open. 2024 Feb 1;7(2):e2354235. doi: 10.1001/jamanetworkopen.2023.54235 (PMC10835513; doi:10.1001/jamanetworkopen.2023.54235)
Supplement: Supplement 1. — eMethods. Cortical Morphometry and Cortical Surface Functioning Preprocessing Workflow eReferences [file jamanetwopen-e2354235-s001.pdf]

Supplemental Online Content

Zuidema TR, Hou J, Kercher KA, et al. Cerebral cortical surface morphology and neural activation pattern among adolescent football players. *JAMA Network Open*. 2024;7(2):e2354235. doi:10.1001/jamanetworkopen.2023.54235

eMethods. Cortical Morphometry and Cortical Surface Functioning Preprocessing Workflow
eReferences

This supplemental material has been provided by the authors to give readers additional information about their work.

© 2024 Zuidema TR et al. *JAMA Network Open.*

eMethods. Cortical Morphometry and Cortical Surface Functioning Preprocessing Workflow

MRI data acquisition

The MRI data were acquired on a 3T Siemens Prisma MRI scanner (Siemens, Erlangen, Germany) equipped with a 64-channel head/neck coil. Cortical morphometry measures were based on high-resolution anatomical images (T1 weighted) acquired using 3D MPRAGE pulse sequence with the following parameters: TR/TE=2400/2.3ms, TI=1060ms, flip angle=8, matrix=320x320, bandwidth=210 Hz/pixel, iPAT=2, which resulted in 0.8 mm isotropic resolution. Resting-state BOLD (blood-oxygen-level-dependent) signal was collected using a simultaneous multislice (SMS) single-shot echo-planar imaging (EPI) sequence with the following parameters: TR/TE=800/30 ms, flip angle=52°, matrix=90×90, FOV=216mm, resolution=2.4 mm isotropic, and multiband acceleration factor=6, with 1000 total volumes acquired over 12 minutes. This sequence was acquired while the participant was asked to relax with their eyes open while passively viewing a crosshair.

Cortical morphometry preprocessing and analyses

The Computational Anatomy Toolbox (CAT12.6; http://www.neuro.uni-jena.de/cat/), which is a plug-in software based on Statistical Parametric Mapping (SPM12, https://www.fil.ion.ucl.ac.uk/spm/software/spm12/), was used for the T1-weighted MRI data preprocessing. The preprocessing consisted of bias-field correction, skull-stripping, and alignment to the Montreal Neurological Institute (MNI) structural template to classify gray matter (GM), white matter (WM), and cerebrospinal fluid (CSF). Spatial normalization was conducted with the Diffeomorphic Anatomical Registration Through Exponentiated Lie Algebra

© 2024 Zuidema TR et al. *JAMA Network Open.*

(DARTEL) registration (1.5 mm).[1] Subsequently, a spherical harmonic method was used to reparametrize the cortical surface mesh based on an algorithm that reduces area distortions to repair any topological defects.[1,2]

Cortical thickness was analyzed based on the workflow specified in the previous study.[3] A voxel-based distance method was used to estimate the WM segment by calculating the distance from the inner GM boundary. Values at the outer GM boundary in the WM distance map was projected back to the inner GM boundary to generate the GM thickness. A central surface was created at the 50% level of the percentage position between the WM distance and GM thickness. For the resultant central surface, a topology correction based on spherical harmonics was used to account for topological defects.[1] Then, the central surface was reparameterized into a common coordinate system through spherical mapping.[4] The cortical thickness data was spatially smoothed with a Gaussian kernel with a 15 mm full-width at half-maximum (FWHM) for this analysis.

The gyrification estimates cortical fold complexity based on spherical harmonics[4] and was calculated as absolute mean curvature.[5] The sulcal depth measures the depth of sulci and is calculated as the Euclidean distance between the central surface and its convex hull based on the spherical harmonics, then transformed with the sqrt function.[5] A 25 mm FWHM Gaussian kernel was used in the spatial smoothing step for gyrification and sulcal depth analyses.

Anatomical data preprocessing for surface-based rs-fMRI analysis

The T1 weighted (T1w) image was corrected for intensity non-uniformity (INU) with N4BiasFieldCorrection,[6] distributed with ANTs 2.3.3,[7] and used as T1w-reference throughout the workflow. The T1w-reference was then skull-stripped with a *Nipype* implementation of the

© 2024 Zuidema TR et al. *JAMA Network Open.*

antsBrainExtraction.sh workflow (from ANTs), using OASIS30ANTs as target template. Brain tissue segmentation of cerebrospinal fluid (CSF), white-matter (WM) and gray-matter (GM) was performed on the brain-extracted T1w using *fast* (FSL 5.0.9, RRID:SCR_002823).[8] Brain surfaces were reconstructed using *recon-all* (FreeSurfer 6.0.1, RRID:SCR_001847),[9] and the brain mask estimated previously was refined with a custom variation of the method to reconcile ANTs-derived and FreeSurfer-derived segmentations of the cortical gray-matter of Mindboggle (RRID:SCR_002438).[10] Volume-based spatial normalization to one standard space (MNI152NLin2009cAsym) was performed through nonlinear registration with antsRegistration (ANTs 2.3.3), using brain-extracted versions of both T1w reference and the T1w template. The following template was selected for spatial normalization: ICBM 152 Nonlinear Asymmetrical template version 2009c [RRID:SCR_008796; TemplateFlow ID: MNI152NLin2009cAsym].[11]

Preprocessing for surface-based rs-fMRI analysis

For each of the 1 BOLD run per participant, the following preprocessing was performed. First, a reference volume and its skull-stripped version were generated using a custom methodology of fMRIPrep. Susceptibility distortion correction (SDC) was omitted. The BOLD reference was then co-registered to the T1w reference using bbregister (FreeSurfer) which implements boundary-based registration.[12] Co-registration was configured with six degrees of freedom. Head-motion parameters with respect to the BOLD reference (transformation matrices, and six corresponding rotation and translation parameters) are estimated before any spatiotemporal filtering using mcflirt (FSL 5.0.9).[13] BOLD runs were slice-time corrected to 0.351s (0.5 of slice acquisition range 0s - 0.703s) using 3dTshift from AFNI 20160207 (RRID:SCR_005927).[14] The BOLD time-series were resampled onto the following surfaces

© 2024 Zuidema TR et al. *JAMA Network Open.*

(FreeSurfer reconstruction nomenclature): fsaverage5. The BOLD time-series (including slice-timing correction when applied) were resampled onto their original, native space by applying the transforms to correct for head-motion. These resampled BOLD time-series is referred to as preprocessed BOLD in the following steps. The BOLD time-series were resampled into standard space, generating a preprocessed BOLD run in MNI152NLin2009cAsym space.

First, a reference volume and its skull-stripped version were generated using a custom methodology of fMRIPrep. Several confounding time-series were calculated based on the preprocessed BOLD: framewise displacement (FD), DVARS and three region-wise global signals. FD was computed using two formulations following Power et al.[15] (absolute sum of relative motions) and Jenkinson et al.[13] (relative root mean square displacement between affines). FD and DVARS were calculated for each functional run, both using their implementations in Nipype.[15] The three global signals were extracted within the CSF, the WM, and the whole-brain masks. Additionally, a set of physiological regressors were extracted to allow for component-based noise correction (CompCor).[16] Principal components were estimated after high-pass filtering the preprocessed BOLD time-series (using a discrete cosine filter with 128s cut-off) for the two CompCor variants: temporal (tCompCor) and anatomical (aCompCor). tCompCor components are then calculated from the top 2% variable voxels within the brain mask. For aCompCor, three probabilistic masks (CSF, WM and combined CSF+WM) are generated in anatomical space. The implementation differs from that of Behzadi et al.[16] in that instead of eroding the masks by 2 pixels on BOLD space, the aCompCor masks were subtracted a mask of pixels that likely contained a volume fraction of GM. This mask is obtained by dilating a GM mask extracted from the FreeSurfer's aseg segmentation, and it ensures that components are not extracted from voxels containing a minimal fraction of GM. Finally, these masks were resampled

© 2024 Zuidema TR et al. *JAMA Network Open.*

into BOLD space and binarized by thresholding at 0.99 (as in the original implementation). Components were also calculated separately within the WM and CSF masks. For each CompCor decomposition, the k components with the largest singular values were retained, such that the retained components' time series were sufficient to explain 50 percent of variance across the nuisance mask (CSF, WM, combined, or temporal). The remaining components were dropped from consideration. The head-motion estimates calculated in the correction step were also placed within the corresponding confounds file. The confound time series derived from head motion estimates, and global signals were expanded with the inclusion of temporal derivatives and quadratic terms for each.[17] Participants with images that exceeded a threshold of 3.0 mm FD were annotated as motion outliers and excluded from the final analysis. All resamplings can be performed with a single interpolation step by composing all the pertinent transformations (i.e. head-motion transform matrices, susceptibility distortion correction when available, and co-registrations to anatomical and output spaces). Non-gridded (surface) resamplings were performed using mri_vol2surf (FreeSurfer).

ALFF and ReHo analyses

After the preprocessing, ALFF and ReHo analyses were conducted using DPABISurf.[18,19] For ALFF, the resampled functional images were spatially smoothed with a full-width-at-half-maximum (FWHM) of 6 mm. De-trend and band-pass filtering (0.01-0.1 Hz) were performed to remove the effects of low-frequency drift and high-frequency noise. The resting-state time series for each voxel was transformed into the frequency domain using a Fourier transform. The square root of the power spectrum was calculated and averaged across 0.01-0.1 Hz within each voxel to obtain a raw ALFF map. The global mean ALFF value was calculated from all voxels across the

© 2024 Zuidema TR et al. *JAMA Network Open.*

whole brain. Finally, ALFF values for each voxel were divided by the global mean ALFF value for standardization. Group comparisons were made for both peak ALFF at specific coordinates and mean ALFF in each brain region.

ReHo analysis was conducted in a similar manner to ALFF.[19] Briefly, after band-pass filtering was performed, surface-based ReHo was calculated by Kendall's coefficient of concordance a given vertex in the surface space with those of its 19 nearest neighbors. This computational approach was repeated for all vertices in surfaces. The individual ReHo maps were smoothened by a 3D Gaussian kernel of 6 mm FWHM for further statistical analysis. Just as in ALFF, group comparisons were made for both peak ReHo at specific coordinates and mean ReHo in each brain region.

Analyses for seed-based rs-FC analysis

After surface preprocessing using the DPABISurf, the following steps took place. First, temporal filtering was applied to the fMRI data, specifically in the frequency range of 0.01 to 0.1 Hz. The mean CSF signal was removed by linear regression. Then, the 3D volumes comprising 210 images for each subject were transformed to the surface. Normalization procedures, including a smoothing operation within the surface space to a 9mm radius, were performed. For the surface mapping of the fMRI data, a weighted-mean method that uses a Gaussian kernel for mapping along the normal was applied. Lastly, regions of interest extraction was performed within the surface space. The bilateral DLPFC was selected a priori as our regions of interest (ROI) because of its involvement in brain injury,[20] as well as its central role in governing diverse cognitive and emotional regulations.[21] The ROI was identified using the HCP-MMP1 (Human Connectome Project Multi-Modal Parcellation version 1.0),[22] and our target region of the

© 2024 Zuidema TR et al. *JAMA Network Open.*

DLPFC was 9-46d, which is located dorsal to the middle frontal sulcus. A Pearson correlation coefficient was generated between DLPFC and the whole brain. The correlation coefficients were then transformed into Fisher *z*-scores for statistical analysis.

eReferences

1. Li Y, Wang N, Wang H, Lv Y, Zou Q, Wang J. Surface-based single-subject morphological brain networks: Effects of morphological index, brain parcellation and similarity measure, sample size-varying stability and test-retest reliability. *Neuroimage*. Jul 15 2021;235:118018. doi:10.1016/j.neuroimage.2021.118018
2. Yotter RA, Dahnke R, Thompson PM, Gaser C. Topological correction of brain surface meshes using spherical harmonics. *Hum Brain Mapp*. Jul 2011;32(7):1109-24. doi:10.1002/hbm.21095
3. Dahnke R, Yotter RA, Gaser C. Cortical thickness and central surface estimation. *Neuroimage*. Jan 15 2013;65:336-48. doi:10.1016/j.neuroimage.2012.09.050
4. Desikan RS, Segonne F, Fischl B, et al. An automated labeling system for subdividing the human cerebral cortex on MRI scans into gyral based regions of interest. *Neuroimage*. Jul 1 2006;31(3):968-80. doi:10.1016/j.neuroimage.2006.01.021
5. Luders E, Thompson PM, Narr KL, Toga AW, Jancke L, Gaser C. A curvature-based approach to estimate local gyrification on the cortical surface. *NeuroImage*. Feb 15 2006;29(4):1224-30. doi:10.1016/j.neuroimage.2005.08.049
6. Tustison NJ, Avants BB, Cook PA, et al. N4ITK: improved N3 bias correction. *IEEE Trans Med Imaging*. Jun 2010;29(6):1310-20. doi:10.1109/TMI.2010.2046908
7. Avants BB, Epstein CL, Grossman M, Gee JC. Symmetric diffeomorphic image registration with cross-correlation: evaluating automated labeling of elderly and neurodegenerative brain. *Med Image Anal*. Feb 2008;12(1):26-41. doi:10.1016/j.media.2007.06.004
8. Zhang Y, Brady M, Smith S. Segmentation of brain MR images through a hidden Markov random field model and the expectation-maximization algorithm. *IEEE Trans Med Imaging*. Jan 2001;20(1):45-57. doi:10.1109/42.906424
9. Dale AM, Fischl B, Sereno MI. Cortical surface-based analysis. I. Segmentation and surface reconstruction. *NeuroImage*. Feb 1999;9(2):179-94. doi:10.1006/nimg.1998.0395
10. Klein A, Ghosh SS, Bao FS, et al. Mindboggling morphometry of human brains. *PLoS Comput Biol*. Feb 2017;13(2):e1005350. doi:10.1371/journal.pcbi.1005350
11. Fonov V, Evans AC, Botteron K, et al. Unbiased average age-appropriate atlases for pediatric studies. *NeuroImage*. Jan 1 2011;54(1):313-27. doi:10.1016/j.neuroimage.2010.07.033
12. Greve DN, Fischl B. Accurate and robust brain image alignment using boundary-based registration. *NeuroImage*. Oct 15 2009;48(1):63-72. doi:10.1016/j.neuroimage.2009.06.060
13. Jenkinson M, Bannister P, Brady M, Smith S. Improved optimization for the robust and accurate linear registration and motion correction of brain images. *NeuroImage*. Oct 2002;17(2):825-41. doi:10.1016/s1053-8119(02)91132-8

© 2024 Zuidema TR et al. *JAMA Network Open.*

14. Cox RW, Hyde JS. Software tools for analysis and visualization of fMRI data. *NMR in biomedicine*. Jun-Aug 1997;10(4-5):171-8. doi:10.1002/(sici)1099-1492(199706/08)10:4/5<171::aid-nbm453>3.0.co;2-l
15. Power JD, Mitra A, Laumann TO, Snyder AZ, Schlaggar BL, Petersen SE. Methods to detect, characterize, and remove motion artifact in resting state fMRI. *NeuroImage*. Jan 1 2014;84:320-41. doi:10.1016/j.neuroimage.2013.08.048
16. Behzadi Y, Restom K, Liau J, Liu TT. A component based noise correction method (CompCor) for BOLD and perfusion based fMRI. *NeuroImage*. Aug 1 2007;37(1):90-101. doi:10.1016/j.neuroimage.2007.04.042
17. Satterthwaite TD, Elliott MA, Gerraty RT, et al. An improved framework for confound regression and filtering for control of motion artifact in the preprocessing of resting-state functional connectivity data. *NeuroImage*. Jan 1 2013;64:240-56. doi:10.1016/j.neuroimage.2012.08.052
18. Yan CG, Wang XD, Lu B. DPABISurf: data processing & analysis for brain imaging on surface. *Science Bulletin*. 2021;66(24):2453-2455.
19. Liu CH, Kung YY, Yeh TC, et al. Differing Spontaneous Brain Activity in Healthy Adults with Two Different Body Constitutions: A Resting-State Functional Magnetic Resonance Imaging Study. *J Clin Med*. Jun 30 2019;8(7)doi:10.3390/jcm8070951
20. Fremont R, Dworkin J, Manoochehri M, Krueger F, Huey E, Grafman J. Damage to the dorsolateral prefrontal cortex is associated with repetitive compulsive behaviors in patients with penetrating brain injury. *BMJ Neurol Open*. 2022;4(1):e000229. doi:10.1136/bmjno-2021-000229
21. Ray RD, Zald DH. Anatomical insights into the interaction of emotion and cognition in the prefrontal cortex. *Neuroscience and biobehavioral reviews*. Jan 2012;36(1):479-501. doi:10.1016/j.neubiorev.2011.08.005
22. Glasser MF, Coalson TS, Robinson EC, et al. A multi-modal parcellation of human cerebral cortex. *Nature*. Aug 11 2016;536(7615):171-178. doi:10.1038/nature18933

© 2024 Zuidema TR et al. *JAMA Network Open.*
