# Supplementary material for: Cerebral Cortical Surface Structure and Neural Activation Pattern Among Adolescent Football Players
Source: JAMA Netw Open. 2024 Feb 1;7(2):e2354235. doi: 10.1001/jamanetworkopen.2023.54235 (PMC10835513; doi:10.1001/jamanetworkopen.2023.54235)
Supplement: Supplement 2. — Data Sharing Statement [file jamanetwopen-e2354235-s002.pdf]

Data Sharing Statement

Zuidema. Cerebral Cortical Surface Morphology and Neural Activation Pattern Among Adolescent Football Players. *JAMA Netw Open*. Published February 01, 2024. doi:10.1001/jamanetworkopen.2023.54235

Data
Data available: No

Additional Information
Explanation for why data not available: There will be no imaging data sharing to public at present time. This is part of an ongoing longitudinal study. When the longitudinal tracking is fully executed, de-identified neuroimaging data will be deposited in public repository (BrainLife).
